# Genome Sequences of *Salmonella enterica* Serotype Typhimurium Blood Clinical Isolate ST4848/06 and Stool Isolate ST1489/06

**DOI:** 10.1128/genomeA.00823-13

**Published:** 2013-10-17

**Authors:** Chi Keung Cheng, Chun Hang Au, Lei Li, Wenyan Nong, Patrick Tik Wan Law, William Man Wai Cheung, Julia Mei Lun Ling, Hoi Shan Kwan

**Affiliations:** School of Life Sciences, Faculty of Science, The Chinese University of Hong Kong, Shatin, Hong Kong SARa; Department of Microbiology, Faculty of Medicine, The Chinese University of Hong Kong, Prince of Wales Hospital, Hong Kong SARb

## Abstract

*Salmonella enterica* serotype Typhimurium human blood strains isolated from outside Africa are rarely sequenced. Here, we report the draft genome sequences of two *S*. Typhimurium clinical strains isolated in the same year, one from blood and another from stool, in order to gain insights into the genetic basis leading to invasive diseases.

## GENOME ANNOUNCEMENT

*Salmonella* food-borne infection is a common but important public health issue worldwide, especially in Hong Kong, as *Salmonella enterica* serovars Typhimurium and Enteritidis have accounted for almost half of the local reported cases of salmonellosis (1). As a result of the continual reductions in the cost of genome sequencing, more and more *S*. Typhimurium genomes are being sequenced. Currently, >200 genome sequences of *S*. Typhimurium strains, either completed or available as sequence contigs or reads, have been deposited in GenBank. Despite *S*. Typhimurium infections usually resulting in self-limiting gastroenteritis, invasive diseases presenting as bacteremia are not uncommon, especially among infants, younger children, and elderly people (2). Among those strains isolated from humans, a majority originated from Africa, and all 117 currently sequenced blood isolates are from Africa as well (3). This lack of sequenced blood isolates outside of Africa has hindered prospective studies of nontyphoidal *Salmonella* (NTS) invasive diseases on other continents. Here, we report the draft genome sequences of two clinical *S*. Typhimurium isolates, ST4848/06 and ST1489/06, the former isolated from blood and the latter isolated from stool in the same year, to facilitate epidemiological studies.

Both strains were isolated from patients admitted to hospitals in the eastern region of Hong Kong in 2006. Genome sequencing was performed on a 454 GS FLX Titanium platform, followed by *de novo* assembly using the Newbler Assembler (4). The Rapid Annotations using Subsystems Technology (RAST) server provided systematic annotation of the draft assembly (5). The assembly resulted in draft genome sequences consisting of 63 contigs of ≥2,038 bp for ST4848/06 and 70 contigs for ST1489/06. Sequencing throughput was 30-fold and 17-fold, respectively. The genomes comprise 4,962,666 bp and 5,007 coding genes for ST4848/06 and 4,762,493 bp and 4,726 coding genes for ST1489/06. Both isolates were shown to be from sequence type 19 (ST19) in multilocus sequence type (MLST) analysis (6), to which most of the sequenced strains isolated from Europe and America belong.

Preliminary bioinformatics analysis indicated that the two isolates show certain genetic variations at both the gene and nucleotide levels. A few single-nucleotide polymorphisms (SNPs) were identified in the *Salmonella* pathogenicity islands (SPIs) and in some of the known virulence factors listed in the Virulence Factors of Pathogenic Bacteria (VFDB) database (7). In addition to the absence of the virulence plasmid pSLT (8), ST1489/06 displays a loss of genetic materials from prophage elements, notably Gifsy-1, Gifsy-2, and Fels-2 (9, 10). In order to randomize genetic variations specific to individual strains, the sequencing of more *S*. Typhimurium human blood strains isolated from outside Africa might open up a new research platform on in-depth comparative genomics analyses and thereby advance our understanding of a potential linkage of genetic elements to nontyphoidal *Salmonella* invasive diseases.

### Nucleotide sequence accession numbers.

The genome nucleotide sequences have been deposited in NCBI GenBank under accession no. AUXE00000000 for *S*. Typhimurium strain ST4848/06 and AUVD00000000 for strain ST1489/06.
